# Early maladaptive schema, attachment style, and parenting style in a clinical population with personality disorder and normal individuals: a discriminant analysis model

**DOI:** 10.1186/s40359-024-01564-5

**Published:** 2024-02-15

**Authors:** Maryam Emami, Maryam Moghadasin, Haniye Mastour, Afshin Tayebi

**Affiliations:** 1 Department of Clinical Psychology, Faculty of Psychology and Educational Sciences, Kharazmi University, Tehran, Iran; 2 Department of Medical Education, School of Medicine, Mashhad University of Medical Sciences, Mashhad, Iran; 3 Department of Psychology, Faculty of Psychology, Islamic Azad University, Karaj, Iran

**Keywords:** Attachment style, Early maladaptive schema, Parenting style, Personality disorders

## Abstract

**Introduction:**

Researchers have shown various variables’ role in forming personality disorders (PD). This study aimed to assess the role of early maladaptive schema (EMS), attachment style (AS), and parenting style (PS) in discriminating between personality disorders and normal individuals.

**Methods:**

In this study, 78 personality disorder patients and 360 healthy volunteers aged 18–84 were selected using convenience sampling. They completed the Schema Questionnaire-Short Form (SQ-SF), Revised Adult Attachment Scale (RAAS), and Baumrind’s Parenting Styles Questionnaire (PSI). Data were analyzed using discriminant analysis with IBM SPSS 25.

**Results:**

The results showed higher mean scores in all early maladaptive schema domains, insecure attachment styles, and authoritarian parenting in the personality disorder group than in the normal group. Also, discriminant analyses revealed that the function was statistically significant and could distinguish between the two groups and a compound of essential variables, disconnection, impaired autonomy, and secure attachment, respectively, discriminating two groups. Given that all components were able to distinguish between the two groups.

**Conclusion:**

Therefore, intervention based on these factors early in life may help reduce the characteristics of personality disorders. Also, considering the role of these factors, treatment protocols can be prepared.

## Introduction

The latest version of the Diagnostic and Statistical Manual of Mental Disorders (DSM-5), which was published in 2015 [1], defines personality disorder (PD) as a “persistent pattern of behaviors and inner experiences that deviate from expectations of the individuals’ culture standards, is inflexible, begins in adolescence or youth, and is fixed over time, lead to dysfunction or functional impairment and is shown in at least 2 of the four domains of cognition, emotion, interpersonal relations or impulse control” [2]. Since the third published edition of DSM, PD has separated from axis one disorder and put onto a separate axis, bringing this area of psychopathology into the researchers’ center of attention [3].

Studies on the prevalence rates of PDs indicate these disorders are common between 9 and 13% in the nonclinical community [4] and 50% in the clinical sample [5]. Prevalence estimates indicate that personality pathology is widespread and has caused public health concerns [3]. Clinical history shows PDs are associated with significant functional impairment [6], considerable risk factors for suicidal behavior [7–9], and are comorbid with a wide range of other psychiatric disorders [7, 10, 11], and social deviation (e.g., crime, addiction) [12, 13].

Accordingly, understanding “How” and “Why” PDs are developed is essential. So far, several studies have been conducted on the description, explanation, and etiology of PDs. There is broad disagreement among scientists about the causes of PDs, but generally, the risk factors for these disorders are multifaceted and influenced by biological, psychological, and social factors [14–16].

Twin and adoption studies have revealed that PDs have a hereditary component; Fontaine and Viding (2008) estimated heritability to be between 30 and 80%, while Buffie (2017) suggested it to be 58% [17, 18]. The remaining unexplained percentage is attributed to environmental influences [19] and adverse childhood experiences [20, 21].

Personality generally arises from the interplay of genetic and environmental factors. Differences in individual traits, characterized under the umbrella term “temperament,” are closely tied to biological processes. These traits, some of which are hereditary, can be observed from early childhood [22, 23]. However, the development of these traits is influenced by both the environment and heredity: children’s experiences shape their character traits, while their traits, in turn, affect their experiences. This ongoing interaction mutually affects psychological processes such as emotional expression, empathy, social behavior, impulse control, and risk-taking [24].

On the other hand, the development of these personality traits is a two-way process: children’s experiences affect the development of their traits, while their traits, in turn, affect the type of experiences they have, and this interaction exists over time [25]. These interactions apply to various psychological processes, such as emotional expression, empathy, attributional style, sociability, impulse control, and risk-taking [24].

Studies investigating the causes of PDs have affirmed the role of genetics [15, 26], early maladaptive schemas (EMS) [27–32], attachment style (AS) [33–36], and parenting style (PS) [37–41].

### Early maladaptive schema (EMS)

Young (1990, 1999) and others hypothesized that these self-defeating emotional and cognitive information-processing patterns might be at the core of many PDs [42]. According to Young, primary maladaptive schemas are “an overarching theme or pattern consisting of memories, emotions, cognitions, bodily sensations, and strong and inflexible beliefs about oneself, the world, and one’s relationships with others that are seen as symptoms in personality disorders.” which the American Psychiatric Association has described [28, 42].

This theory suggests negative/traumatic childhood or adolescence experiences are the primary source of these dysfunctional patterns, perpetuated throughout the lifespan, and generate self-defeating behavioral patterns [43]. The activation of primary maladaptive schemas causes intense emotions and frustrations, which show that their basic emotional needs are not satisfied, and the symptoms observed in personality disorders are the methods that people use to cope with this issue [28]. EMSs are assumed to be highly stable and resistant to change, acting like templates to assign repetitive meanings to the individual’s experiences and guide their thoughts, perceptions, emotions, and behavior [31]. Findings indicate EMSs are dysfunctional to a significant degree [42] and lead to emotional, interpersonal, and professional relationship confusion and dissatisfaction, psychological distress, and PDs [42, 44].

EMSs are believed to underlie, perpetuate, and maintain characteristics commonly seen in PDs. So far, clinical and nonclinical studies have investigated the relationship between EMSs and PDs. These research studies have generally indicated that EMSs contributed significantly to the prediction of PDs [28, 45], there are significantly higher levels of EMSs in PDs in comparison to the control group and other patients [27, 30, 46], and some EMSs predict certain PDs [28–31, 47, 48]. Also, some of these research studies found a relationship between the PD and EMS domain [27, 31, 47, 49]. For example [47] in their research indicated some domains of EMSs relate to a broader vulnerability factor for PDs (i.e., disconnection and rejection for both borderline and avoidant PDs), and some domains differentially relate to the specific PDs (i.e., other-directedness domain for dependent personality disorder and over vigilance for obsessive-compulsive personality disorder). Narcissistic personality disorder was positively associated with impaired limits [31, 49], disconnection/rejection, vigilance, and inhibition [31] and negatively associated with the other-directedness domain. Paranoid personality disorder was associated with the disconnection and rejection and the impaired autonomy and performance domain, and borderline and antisocial personality disorder were associated with the disconnection and rejection and the impaired limits domain [49]. Antisocial personality disorder was related to three EMS domains: impaired limits, impaired autonomy/performance and disconnection/rejection, and borderline personality disorder was related to all domains except other-directed [27].

### Attachment

Another theory that has been described as a conceptual framework for understanding PDs is attachment theory (AS) [34]. This theory was formulated by British psychoanalyst John Bowlby (1969, 1973, 1980) and American-Canadian developmental psychologist Mary Ainsworth (1991). This theory explains how the emotional bonds one forms with primary caregivers during infancy have lasting effects and extend into adulthood, serving as enduring templates to determine their characteristic ways of relating to others [35, 50, 51]. In other words, the attachment theory suggests that how people relate to others and respond to intimacy throughout life are learned during infancy through interactions with primary caregivers or “attachment figures.” [34, 35].

Bowlby (1969–1999) claimed infants internalize their interactions with primary caregivers as a cognitive framework that comprises mental representations of the world, self, and others. Such representations are called “internal working models.” They are hypothesized to shape the development of ASs [33] and to establish a base for personality development, identity formation, subsequent intimate relationships [35, 52], expectations about interpersonal relations, social acceptance, as well as attitudes to rejection, strategies for coping with distress, and mental health throughout life [34, 35, 52].

Attachment theory has proven to be a fruitful framework for predicting vulnerability to psychopathology. Bowlby (1973, 1980) postulated that the development of the negative representation of self or others during the early years of life can be related to the subsequent development of psychopathology [53]. Many personality theorists have considered attachment theory as a framework for understanding the development, maintenance, and treatment of the interpersonal difficulties and adaptations that characterize personality pathology [34, 35, 54].

PDs are characterized by distorted representations of self and others, as well as dysfunctional interpersonal relationships [36]. According to attachment theory, PDs reflect insecure internal working models that are assumed to operate in such a way as to confirm or fulfill themselves. These representational working models, in this way, make themselves rigid and inflexible and cause difficulties in social, occupational, and relational functioning [54].

Further, a large body of empirical research has examined the relationship between PDs and ASs and supported the general role of insecure attachment in forming PDs [33, 55–57]. Along the same lines, research studies have shown that secure attachment negatively predicts PD [58, 59].

Some research has studied the relationship between attachment and PDs across three different clusters of PDs and provided evidence of the role of attachment in Cluster B PDs [56, 60]. Cascio and Alaimo indicated that preoccupied attachment is a specific feature of patients’ Cluster C PDs, and Cluster A and B disorders were associated with fearful avoidant attachment [61].

### Parenting style (PS)

The third variable hypothesized in this study to act as a factor influencing the development of PDs is parenting style (PS). Many researchers consider the interactions between the individual and their environment as the most critical factor in the development of PDs [62, 63]. Prominent research and theory have considered family processes as significant sources of socialization for children and the development of personality [64, 65]. Along the same lines, research indicates children brought up by warm parents tend to show better social adjustment [66].

As dysfunctional interpersonal relationships comprise a core feature of PDs [39], it’s probably safe to argue that socialization deficits observed among individuals with PD might partly emerge from problematic parenting [64]. One theory to conceptualize the impact of early socialization on the development of PDs has been Diana Baumrind’s PSs (1967, 1978, 1991). She conceptualized PSs along two dimensions: parental demand (e.g., control) and parental response (e.g., warmth). Accordingly, Baumrind defined three PSs: authoritative (high demand and high responsiveness), authoritarian (high demand and low responsiveness), and permissive (low demand and high responsiveness) [67].

Research on PSs suggested lack of parental care, overprotection, or both in childhood resulted in a change in normal personality, such as higher neuroticism, self-criticism, and perfectionism [68].

Many studies have found associations between parenting and PDs [37, 41, 64, 69–72]. For example, Cheng et al. [41] showed parental rejection and over-protection were linked to the higher occurrence of PD, while emotional warmth was negatively related to PD. In addition, it has been concluded that lower levels of care and higher levels of overprotection characterize patients with PDs. Similarly, personality-disordered patients perceived less parental care and more paternal freedom control and autonomy denial than normal adolescents and adults [40, 41].

Nordahl et al. [30] declared patients with antisocial, schizoid, and schizotypal PDs did not show any significant association with any of the EMSs. Also, in this study, the scores of enmeshment/underdeveloped self-schemas, emotional deprivation, and entitlement did not differentiate between patients with PD and without a diagnosis of PD.

Research also indicates a significant relationship between AS, EMS, and perceived PS. The interaction between a child’s unique nature, temperament, and negative experiences like maladaptive PSs contributes to the development and persistence of maladaptive schemas [42, 71, 73]. Studies have shown that negative parenting practices from both parents are linked to stronger levels of schema domains [74, 75]. ASs are also shaped by genetic factors, a child’s temperament, and attachment experiences [76, 77]. Some studies have found a positive relationship between authoritative parenting and secure attachment and negligent and authoritarian parenting, which predicted avoidant attachment [78]. The findings of a meta-analysis indicated a strong positive association between insecure attachment styles (anxious and avoidant) and primary maladaptive schemas. In contrast, secure attachment negatively correlates with primary maladaptive schemas [79].

In this study investigating the origins of PDs, the focus was on three key factors: schema, attachment, and parenting. Based on previous research, these variables have shown significant relevance to understanding PDs. However, despite this background, it remains unclear which factors significantly influence the development of these disorders. Therefore, the current study aimed to examine all three variables simultaneously and determine their respective contributions in distinguishing individuals with PDs from those without.

One of the innovative aspects of this study is looking into how the linear relationships between these constructs could be used to discriminate the normal (NR) group from the disordered group. In light of the data declared in the current study, this research aims *to (1)* Determine whether PDs can be predicted based on EMSs, AS, and perceived PS; *(2)* Can variables differentiate between the NR and PD groups? and *(3)* Another purpose of this study was to determine which component or components could demonstrate such distinction and estimate each variable’s share in the difference between the two groups.

## Materials and methods

### Participants and procedure

The study included two groups: normal and clinical. The clinical group included outpatients seeking treatment at an Iranian Specialized Psychiatry clinic in Tehran and Alborz provinces. Researchers visited three psychiatric clinics in person to collect samples from individuals with PDs. They talked to individuals diagnosed with PD by a psychiatrist, and after giving informed consent to all patients, they filled out a paper and online questionnaire. Inclusion criteria were *(a)* a primary diagnosis of PDs by a clinical psychologist and psychiatrist, *(b)* older than 18 and younger than 84 years old, and *(c)* a level of education of at least middle school. The final sample comprises 78 outpatients with PDs (Female *N* = 30, 61.5%; with mean age = 32; Male *N* = 38, 38.5%; with mean age = 32). Patients were diagnosed with PD (clusters A (*N* = 19), B (*N* = 17), and C (*N* = 42)) according to the (DSM-5, American Psychiatric Association, 2013).

In discriminant analysis, the smallest group should have more individuals than the number of predictor variables [80]. This study’s smallest group consists of individuals with PDs, totaling 78 participants, while we had 11 predictor variables. Hence, the number of individuals in the personality-disordered group was five times greater than the total number of variables, confirming the hypothesis.

In this study normal population, including 422 subjects, was selected by convenience sampling. The examiner evaluated them after screening with SCID, ensuring that all entry criteria were met and conducting thorough interviews with the participants. As a result, 62 subjects were excluded due to not meeting the entry criteria. Ultimately, data from 360 normal subjects were analyzed. Participants who were willing to participate received and filled out the questionnaire online. Inclusion criteria were *(a)* no PD and other psychiatric disorders, *(b)* older than 18 and younger than 84, and *(c)* level of education of at least middle school. The final sample comprised 360 (Female *N* = 210, 58.3%; with mean age = 29; Male *N* = 150, 41.7%; with mean age = 30). The gender frequency difference between the two groups was evaluated, and no significant difference was observed in terms of this variable between the two groups (*X*^*2*^ = 0.272, *df* = 1, *P* = 0.602).

### Instrument and questionnaire

#### The Schema Questionnaire-Short Form (YSQ-SF)

It is a 75-item self-report assessing 15 different EMSs that are clustered in five domains, including *(a)* disconnection and rejection, *(b)* impaired autonomy and performance, *(c)* impaired limits, *(d)* other-directedness, and *(e)* over-vigilance and inhibition. Each item is rated on a 6-point Likert scale ranging from 1 (Absolutely wrong) to 6 (Absolutely true). The average score of each schema is calculated by summing the outcomes of all related items and then dividing this by the total number of questions. Evidence supports this instrument’s reliability, validity, and factor structure of the SQ-SF [81, 82]. The Persian version of the YSQ-SF has demonstrated adequate validity and reliability in Iranian samples [83, 84]. For all subscales, Cronbach’s αs for the YSQ‐SF subscales were estimated between 0.69 and 0.90 [83].

#### Revised Adult Attachment Scale (RAAS)

It is an 18-item self-report instrument that includes three subscales (each with six items): *(a)* comfort with closeness, *(b)* comfort with depending on others, and *(c)* anxious concern about abandonment. According to these subscales, three AS of secure, anxious, and avoidant are identified. Each item is rated on a 5-point Likert scale ranging from 1 (I completely agree) to 5 (I completely disagree). Internal consistency reliability, α coefficient, and retest reliability after a 2-month interval were 0.68, 0.71, and 0.52 for close, dependency, and anxiety subscales, respectively [85, 86]. Collins and Read (1996) reported adequate internal consistency. Cronbach’s alphas for the close, depend, and anxiety subscales were 0.77, 0.78, and 0.85, respectively. Research on the Iranian population indicates a high validity of 0.95 for this variable. Moreover, Cronbach’s alpha for this scale is reported to be 0.8, further underlining its high-reliability coefficient [87].

#### PS Inventory (PSI)

This questionnaire was designed by Diana Baumrind in 1972, and it includes 30 items that evaluate three PSs: authoritative, authoritarian, and permissive styles. The question’s response pattern follows a 5-point Likert scale from “Absolutely opposed” to “Absolutely agree.” The reliability of this questionnaire, by test-retest method, is 0.81 for permissive, 0.92 for authoritarian, and 0.92 for authoritative PS [88]. In evaluating the validity of this instrument, the relationship between permissive and authoritarian has been reported as -0.50, and between authoritative and authoritarian, -0.52 [89]. In Iran, Esfandiari (1995) has declared the reliability of subscales by test-retest, 0.69 for permissive style, 0.77 for authoritarian, and 0.73 for authoritative [90].

### Statistical analyses

Participants were assessed using YSQ-SF, RAAS, and PSI. According to the nature of the variables, discriminant analysis was used to analyze the data to predict group membership in dependent variable classes from a set of independent variables.

First, the hypotheses of diagnostic analysis were examined. One of the assumptions for discriminant analysis is the lack of a multi-collinearity relationship between independent variables. The correlation between independent variables was first obtained in this study to conduct the discriminant analysis. Calculating the matrix of average correlations within groups indicated the lack of a multi-collinearity relationship. Also, log determinants and Box’s M test (*F* = 1.121, *P* = 0.235) showed that the variance/covariance matrix of the two considered groups is equal. Since all the discriminant analysis test assumptions have been confirmed, this study used the discriminant analysis test to examine the research assumptions. All statistical analyses were performed using SPSS (version 25.0).

## Results

From among 438 participants (258 females and 180 males) in this research, 78 were included in the PD group (17.8%) and 360 in the NR group (82.2%). Table 1 shows the demographic characteristics of the participants in the two groups. The average age was 32 (SD = 6.9) and 29 (SD = 8) for the PD and NR groups, respectively. The PD group comprised 19 people with Cluster A (24.4%), 17 people with Cluster B (21.8%), and 42 people with Cluster C (53.8%).

Pearson correlation matrix for the relationship between schema domain, AS, and PS in PD and NR individuals has been provided in Table 2. Additionally, Table 3 presents the descriptive statistics of the subjects. According to this table, compared to the NR group, people with PDs scored higher on average in all five EMS domains: anxious AS, avoidant AS, and authoritarian PS. The average score for the NR group in secure AS, authoritative PS, and permissive PS was higher than the PD group.

The conducted discriminant analysis was a two-group analysis, so one discriminant function was performed (Table 4). The discriminant function was significant as Wilks` lambda was 0.860 (*P* < 0.001). This value indicated the existence of differences between groups (Table 5).

**Fig. 1:**
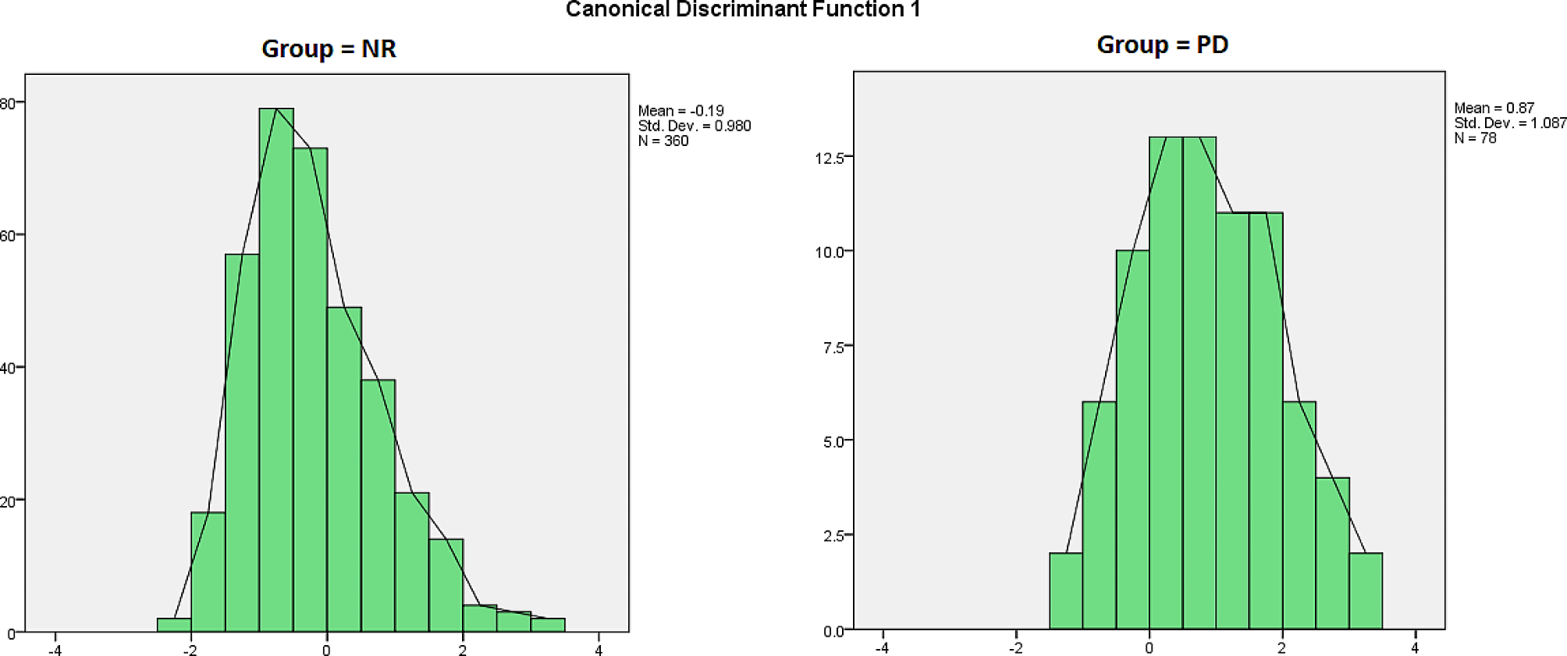
The plot of the discriminant function for NR and PD groups

Table 6 shows standardized coefficients and structure matrix. Canonical discriminant function coefficients were used to evaluate the independent variable’s unique contribution to the discriminant function (Fig. 1). The canonical structure matrix revealed the correlations between each independent variable and the discriminant functions. It allowed us to compare correlations and see how closely a variable was related to each function [91].

Structure matrix coefficients value showed PD was mainly determined by positive relation discriminant functions with disconnection/rejection domain (0.844), impaired autonomy/performance (0.795) schema domain, and negative relation discriminant functions with secure AS (-0.651), respectively (Table 6). The classification result clearly showed how constituting the sample was distributed across groups. According to the results, 261 individuals (72.5%) of the NR group were classified correctly. Also, 53 individuals (67.9%) in the PD group were correctly classified. A total of 71.7% of individuals were distributed in line with the expected classification (Table 7).

**Table 1:** Demographic characteristics of participants

		Gender		Age
Group	**Mean**	**Frequency**	**Percent**	**Mean**
NR	Male	150	41.7	30.46
	Female	210	58.3	29.19
	Total	360	100.0	
PD	Male	30	38.5	32.80
	Female	48	61.5	32.22
	Total	78	100.0	

NR: Normal

**Table 2:** Pearson correlation matrix for the relationship between schema domain, AS, and PS in PD and NR groups

Variables	DR	IP	II	OD	OI	ATI	ARI	PM	SC	AV	AX
PD Group (*N* = 78)											
DR	1										
IP	0.69^**^	1									
II	0.54^**^	0.40^**^	1								
OD	0.55^**^	0.47^**^	0.21	1							
OI	0.49^**^	0.34^**^	0.39^**^	0.42^**^	1						
ATI	− 0.13	− 0.17	0.00	0.12	− 0.02	1					
ARI	0.49^**^	0.32^**^	0.25^*^	0.24^*^	0.14	− 0.47^**^	1				
PM	− 0.40^**^	− 0.30^**^	− 0.12	− 0.20	− 0.08	0.60^**^	− 0.63^**^	1			
SC	− 0.21	− 0.19	− 0.31^**^	0.18	− 0.27^*^	0.10	0.01	0.08	1		
AV	0.35^**^	0.14	0.41^**^	0.13	0.33^**^	− 0.12	0.27^*^	− 0.18	− 0.28^*^	1	
AX	0.63^**^	0.50^**^	0.49^**^	0.39^**^	0.46^**^	− 0.03	0.30^**^	− 0.17	− 0.18	0.24^*^	1
NR Group (*N* = 360)											
DR	1										
IP	0.69^**^	1									
II	0.59^**^	0.49^**^	1								
OD	0.59^**^	0.62^**^	0.45^**^	1							
OI	0.47^**^	0.43^**^	0.44^**^	0.51^**^	1						
ATI	− 0.20^**^	− 0.11^*^	− 0.03	− 0.05	− 0.03	1					
ARI	0.47^**^	0.36^**^	0.32^**^	0.32^**^	0.32^**^	− 0.50^**^	1				
PM	− 0.35^**^	− 0.17^**^	− 0.19^**^	− 0.08	− 0.09	0.62^**^	− 0.62^**^	1			
SC	− 0.37^**^	− 0.31^**^	− 0.30^**^	− 0.12^*^	− 0.33^**^	0.14^**^	− 0.23^**^	0.26^**^	1		
AV	0.42^**^	0.21^**^	0.32^**^	0.20^**^	0.35^**^	− 0.08	0.26^**^	− 0.21^**^	− 0.32^**^	1	
AX	0.66^**^	0.49^**^	0.42^**^	0.50^**^	0.43^**^	− 0.11^*^	0.30^**^	− 0.18^**^	− 0.33^**^	0.33^**^	1

NR: Normal, DR: Disconnection/rejection schema domain, IP: Impaired autonomy/performance schema domain, II: Impaired limits schema domain, OD: Other-directedness schema domain, OI: Over vigilance/inhibition schema domain, SC: Secure AS, AV: Avoidant AS, AX: Anxious AS, ATI: Authoritative PS, ARI: Authoritarian PS, PM: Permissive PS.

*p-value < 0.05, and **p-value < 0.01

**Table 3:** Descriptive statistics (Mean and Standard Deviation) for NR and PD groups

		Group			
		NR		PD	
		M	SD	M	SD
EMS	Disconnection/rejection	57.67	20.21	76.26	23.82
	Impaired autonomy/performance	36.86	15.24	49.92	17.08
	Impaired limits	29.56	8.36	34.44	9.61
	Other-directedness	26.53	8.44	30.68	10.08
	Over vigilance/inhibition	31.47	9.23	36.61	9.35
AS	Secure	16.18	4.29	13.20	4.57
	Avoidant	13.82	3.85	15.11	3.32
	Anxious	10.33	5.78	13.35	5.95
PS	Authoritative	31.94	8.50	28.34	8.23
	Authoritarian	25.83	8.39	29.94	7.87
	Permissive	25.02	6.06	22.67	5.96

NR: Normal, M: Mean, SD: Standard deviation

**Table 4:** Tests of equality of group mean

	Wilks’ Lambda	F	df1	df2	P
Disconnection/rejection	0.896	50.75	1	436	0.001
Impaired Autonomy/performance	0.906	45.00	1	436	0.001
Impaired limits	0.955	20.63	1	436	0.001
Other-directedness	0.968	14.40	1	436	0.001
Over vigilance/inhibition	0.957	19.77	1	436	0.001
Secure	0.935	30.17	1	436	0.001
Avoidant	0.983	7.51	1	436	0.006
Anxious	0.962	17.39	1	436	0.001
Authoritative	0.974	11.58	1	436	0.001
Authoritarian	0.965	15.75	1	436	0.001
Permissive	0.978	9.65	1	436	0.002

**Table 5:** Wilks’ lambda summary of canonical discriminant functions

Function	Eigenvalue	% of variance	Cumulative %	Canonical correlation	Wilks’ Lambda	Chi-square	P
1	.163^a^	100.0	100.0	0.375	0.860	65.166	0.001

**Table 6:** Structure matrix and canonical discriminant function coefficients

	Canonical discriminant function coefficients		Structure matrix
	Function		Function
	1		1
Disconnection/rejection	0.586		0.844
Impaired autonomy/performance	0.381		0.795
Impaired limits	0.008		0.538
Other-directedness	− 0.087		0.450
Over vigilance/inhibition	0.118		0.527
Secure	− 0.375		− 0.651
Avoidant	− 0.080		0.325
Anxious	− 0.188		0.494
Authoritative	0.046		− 0.403
Authoritarian	− 0.208		0.470
Permissive	− 0.223		− 0.368

**Table 7:** Classification Results^a,c^

			Predicted group membership		Total
			NR	PD	
Original	Count	NR	261	99	360
		PD	25	53	78
	%	NR	72.5	27.5	100.0
		PD	32.1	67.9	100.0
Cross-validated^b^	Count	NR	256	104	360
		PD	29	49	78
	%	NR	71.1	28.9	100.0
		PD	37.2	62.8	100.0

NR: Normal, PD: personality disorder, (a) 71.7% of original grouped cases correctly classified. (b) Cross-validation is done only for those cases in the analysis. In cross-validation, each case is classified by the functions derived from all cases other than that; (c) 69.6% of cross-validated grouped cases are correctly classified

## Discussion

In response to the questions raised in this study, the results showed that the EMS, AS, and PS variables could predict personality disorders. These variables can be used to identify individuals at risk for developing PDs. Furthermore, the variables were also found to differentiate between the NR group and those with PDs, so they can be used to distinguish between individuals with a normal personality and those with a PD. Additionally, the study found that specific components contributed more to differentiating between the two groups. The components of schema domains involving disconnection/rejection, impaired autonomy/performance, and secure attachment style were found to have the highest contribution in creating this distinction.

The results of this study indicated ASs were capable of discriminating between NR and PD groups. Secure attachment, insecure-anxious attachment, and insecure-avoidant ASs made the highest distinction between the two groups, respectively. In this research, consistent with the findings of some other studies, secure attachment negatively predicted PD [58, 59]. Moreover, the PD group differed from the NR group concerning anxious and avoidant attachments. In the current study, as the other studies declared, they played a more significant role in discriminating between the two groups [58, 92].

Another finding revealed ASs could predict PDs, with secure attachment being a negative predictor and anxious and avoidant insecure attachments being a positive predictor for PDs, in which some studies obtained the same results [33, 34, 57].

PDs are characterized by enduring maladaptive patterns of behavior, which include interpersonal (disturbances in arousal regulation or impulse and emotion control), intrapersonal (incompetent relationship patterns), and social components (which create conflict with others and the social institutions) [33]. The attachment theory could explain such characteristics of PDs. Each PD has a distinctive and incompetent intrapersonal style that is usually the characteristic feature of the disorder. There is also a series of experimental supports for the hypothesis that PD is a disorder in intrapersonal relationships [34, 54]. A study by Meyer, et al. has found that dysfunctional intrapersonal behaviors make up 45% of diagnostic criteria for the PDs in DSM-IV, outstripping the remaining criteria [dysfunctional cognition (23%), emotional disorders (20%), and the other behaviors (12%)] [59]. The formation of secure attachment, a main growth stage, is influenced by how the children interact with their caregivers. Since the mental representations of the type of primary relationships extend to an individual’s future and affect their relationships, it may explain how an insecure attachment damages the intrapersonal relationships of an individual with PD.

The findings indicate that PSs can discriminate between the NR and PD groups. In this study, the authoritarian PS positively correlated with the PDs, whereas the authoritative PS (characterized by high responsiveness, high care, and high demands) negatively correlated with the PDs. The results are consistent with the studies reporting that patients with PD receive lower parental care but higher freedom, control, and autonomy denial (the characteristics of authoritarian parenting) than normal adolescents and adults [55–57]. As mentioned before, one of the main features of PDs is a significant problem in intrapersonal relationships. How a child behaves generally depends on the PSs that the parents adopted. A good relationship with parents would help the children achieve social and emotional adjustment during growth [64, 65].

Furthermore, the parents affect the socialization process and impact various components of personality, such as children’s cognition, emotion, and behavior [93]. People with PD lack at least two of the four areas of intrapersonal relationship: cognition, emotion, and impulse control [5]. Our hypothesis was confirmed by the studies that have indicated that problematic parenting increases the risk of developing PD [41, 64, 69, 71, 94].

Apart from authoritative parenting, permissive parenting also negatively correlated with the PD group in this study. The permissive PS, characterized by a high level of care, may serve as a protective factor against such PD traits. Limited research has explored the association between the perceived parenting style within the PD group and how it differs from the NR group. However, the findings have shown that A review of the conclusions of research such as Henschel, Claudio, and Kılıçkaya on the effect of permissive PS on the development of PDs revealed that narcissistic PD is related to permissive parenting [95–97]. Moreover, Morrison’s study showed that PSs that lack boundaries are over-indulgent or inconsistent and may predispose children to develop histrionic PD [98], which resembles permissive PS. The results of research on permissive PS require further analysis.

Among the ten specific PDs, the schizoid, schizotypal, paranoid, borderline, avoidant, and obsessive-compulsive disorders are characterized by features that indicate a record of parental neglect and lack of adequate care for children [20, 99–101].

The findings indicate distinctions between the PD and the NR groups in the disconnection and rejection, impaired autonomy and performance, impaired limits, over-vigilance and inhibition, and other-directedness domains, respectively.

In this study, the scores of EMSs in PD individuals were higher than in the NR group, and the finding of this study was supported by the studies that show some early maladaptive schemas were predictors of PDs [27–31, 47, 102, 103]. People with EMSs cannot perceive and analyze the information, happenings, relationships, and their surroundings as they exist. They perceive the situation distortedly and react to it based on the pre-existing beliefs in their schemas. They also have low flexibility and cannot accept that this is, in fact, their schemas that create problems, not the people around them or their experiences. Schemas also cause problems in an individual’s cognitive and emotional aspects, feelings, identity, intrapersonal relationships, as well as occupational and social performance, which closely resemble the qualities of people with PD. The problems of people with PD could be attributed to the maladaptive schemas that they have developed.

Findings indicated all components could significantly separate the NR and PD groups. The following variables accounted for the most significant share in making a distinction among the two groups respectively: (1) Disconnection and rejection, (2) Impaired autonomy and performance, (3) Secure attachment, (4) Impaired limits, (5) Over vigilance and inhibition, (6) Anxious attachment, (7) Authoritarian parenting, (8) Other-directedness, 9. Authoritative parenting, 10. Permissive parenting, and 11) Avoidance attachment.

Disconnection and rejection include abandonment/instability, mistrust/abuse, emotional deprivation, defectiveness/shame, and social isolation/alienation schemas. Patients with these schemas cannot experience secure attachment to others and have grown up in a family characterized as rejectionist, unstable, blameful and humiliating, abusive, or isolated. These people’s basic needs for security, tranquility, acceptance, support, and sympathy have not been satisfied. Meanwhile, among the EMS, patients with schemas in this domain had traumatic childhoods and were often the most damaged. In adults, they had unhealthy relationships [42]. According to the explanations, the domain of disconnection and rejection includes schemas with features that cover some of the concepts related to insecure attachments (anxiety and avoidance) that, in adults, had unhealthy relationships.

Impaired autonomy and performance domains include dependence/incompetence, vulnerability to harm or illness, enmeshment/underdeveloped self, and failure schema. Patients with these schemas have grown up in families that have been entangled, overprotective, rigid, and lacking self-confidence, hindering their independence and readiness to live outside the family and perform well [42]. Moreover, impaired autonomy and performance include characteristics that could explain problematic parenting, especially authoritarian parenting.

### Limitations

Limitations of the study include different size numbers of the normal and disordered groups. Due to the challenges posed by individuals with personality disorders regarding cooperation and healthy social interaction, it becomes challenging to expand the size of the personality disorder group. This expansion requires a lengthy collection period for obtaining samples and the cooperation and support of additional treatment centers such as hospitals and psychiatric clinics. Meanwhile, a personality disorder is comorbid with other psychiatric disorders. The relation between these variables should be studied through a modeling research method.

## Conclusions

The current study declared that, compared to normal individuals, PD patients had a higher level of EMS domain (e.g., disconnection and rejection and impaired autonomy), insecure attachment, and authoritarian perceived PS. The highest level of distinction was caused by disconnection and rejection domain schema, impaired autonomy and performance, secure attachment, impaired limits domain, over vigilance and inhibition, anxious attachment, authoritative parenting, other-directedness domain schema, authoritarian parenting, permissive parenting, and avoidant attachment, respectively. So, the result suggests that special attention should be paid to these factors in the treatment and psychopathology of PD.

## Data Availability

The datasets used and/or analyzed during the current study are available from the corresponding authors upon reasonable request.
